# Extracorporeal Photopheresis in Lung Transplantation: Present Applications and Emerging Research

**DOI:** 10.1097/TXD.0000000000001831

**Published:** 2025-09-02

**Authors:** Sophia Alemanno, Peter Jaksch, Alberto Benazzo

**Affiliations:** 1 Vienna Lung Transplant Program, Department of Thoracic Surgery, Medical University of Vienna, Vienna, Austria.

## Abstract

Extracorporeal photopheresis (ECP) is an immunomodulatory therapy currently used as an add-on treatment for the prevention and management of organ rejection in lung transplantation. Thanks to its immunomodulatory properties and its ability to reduce the need for immunosuppressive therapies, ECP presents a promising therapeutic option, especially for high-risk patients with comorbidities, infections, or malignancies. This review provides a comprehensive overview of the current indications, clinical experience, and ongoing research surrounding the use of ECP in lung transplantation. Additionally, it delves into the current understanding of the mechanism of action of ECP, its potential role in lung transplantation, and the limitations identified in existing studies. By highlighting these aspects, the review aims to lay the groundwork for future research, which could further elucidate the mechanisms underlying this promising therapy and contribute to the standardization of therapeutic protocols.

## Graphical abstract

**Figure:**
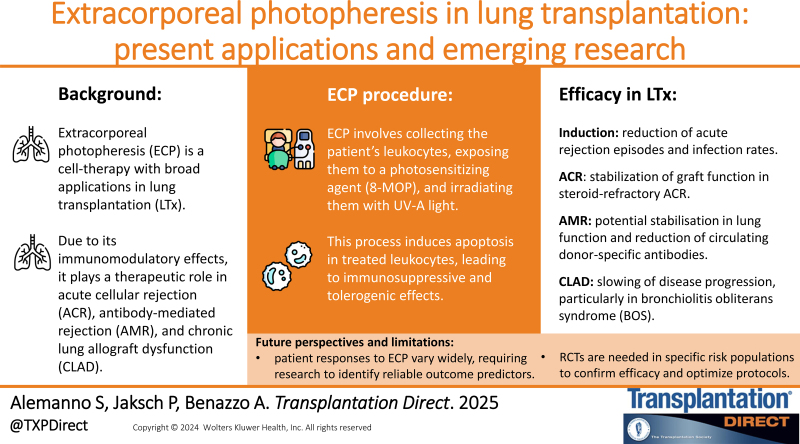

Lung transplantation (LTx) is a widely recognized, life-saving procedure for patients with end-stage respiratory failure.^1^ Despite recent advancements, long-term survival remains notably lower compared with other organ transplants. Data from the US Organ Procurement and Transplantation Network show that survival rates for LTx recipients are 85% after 1 y and 55% after 5 y.^2^ The limited average life expectancy seen after LTx is largely attributed to chronic rejection, namely chronic lung allograft dysfunction (CLAD).^3^ Furthermore, acute rejection, both cellular and antibody-mediated, contributes to poor short-term graft survival and represents important risk factors for the development of CLAD.^4-9^ Finally, de novo donor-specific antibodies (dnDSAs) have been associated with lower survival rates and a higher incidence of antibody-mediated rejection (AMR).^4-9^ Due to the constant exposure to the environment, pulmonary grafts are more susceptible to both recurrent infections and chronic inflammatory stimuli, which promote continuous antigenic exposure and contribute to chronic immune activation.^10^ LTx recipients typically receive various immunosuppressive regimens throughout their lives. These include induction therapy, often based on antilymphocyte antibodies such as antithymocyte globulin (ATG), monoclonal anti-CD3 antibodies, or monoclonal antibodies targeting the interleukin-2 receptor. Maintenance therapy usually consists of a combination of 3 drugs: a calcineurin inhibitor, cyclosporine A or tacrolimus, a cell-cycle inhibitor, and corticosteroids.^11^ In cases of acute rejection, therapy is typically intensified. Although these immunosuppressive strategies are effective in reducing the risk of acute rejection, they often fail to significantly improve long-term lung function. Moreover, they are frequently associated with serious adverse effects, including increased susceptibility to opportunistic infections—such as bacterial, viral (notably cytomegalovirus), and fungal infections—as well as toxicities ranging from bone marrow suppression and organ dysfunction to an elevated risk of malignancies.^10^ As a result, there is a pressing need for novel therapeutic strategies that offer more effective ways to address the host immune response. Ideally, such treatments should provide targeted immune modulation, focusing on donor-specific immunity, thereby reducing the need for broad immunosuppression and minimizing risks of infection.^12^ Due to its safe side-effect profile and immunomodulatory properties, extracorporeal photopheresis (ECP) has emerged as a promising therapeutic option in LTx.

## ECP PROCEDURE

ECP involves 2 consecutive key processes within a closed-loop sterile system: leukapheresis and photoactivation.^13,14^ First, peripheral blood is drawn, and mononuclear cells (MNCs) are separated from the whole blood via centrifugation. The collected MNCs in the so-called buffy coat bag are then treated with 8-methoxypsoralen (8-MOP) solution and exposed to ultraviolet A (UVA) light extracorporeally, triggering apoptosis.^15^ Once treated, the MNCs—now called photopheresates—are returned to the patient through intravenous infusion.

In our institution, ECP is delivered in 2-d treatment cycles using the THERAKOS CELLEX Photopheresis System (Mallinckrodt Pharmaceuticals, Hazelwood, MI) through either double- or single-needle access.^16^ Other protocols foresee a 1-d cycle or offline devices. During each leukapheresis procedure, approximately 1500 mL of whole blood is processed to isolate peripheral blood MNCs by centrifugation, which are collected in the buffy coat.^16^ To the MNC collection bag, 8-MOP (Uvadex; Mallinckrodt Pharmaceuticals) is added, which is then irradiated with UVA light. After photoactivation, the buffy coat is reinfused into the patient. Each complete procedure lasts approximately 2 h.^16^

ECP remains a challenging therapy from a logistical point of view. In the majority of cases, it is performed in tertiary hospitals, and the availability of this technology remains limited. Due to the duration of the procedure and the need for venous access, patient adherence is between 60% and 80%. Side effects such as nausea, vomiting, and circulatory instability have become rare, thanks to the improvement of ECP technology and the use of intravenous psoralen.^17^ Absolute contraindications to ECP include hypersensitivity to psoralen, aphakia, pregnancy or lactation, and severe cardiac disease; relative contraindications include thrombocytopenia, arterial hypotension, congestive heart failure, and low hematocrit levels.^17^

## MECHANISM OF ACTION

Although the precise mechanisms underlying ECP are not fully elucidated, 3 main immunological processes are consistently described: (1) induction of apoptosis by 8-MOP/UVA exposure, (2) monocyte differentiation into dendritic cells (DCs), and (3) modulation of cytokine profile and T-cell subsets.^18^ DNA crosslinking induced by UVA-activated 8-MOP triggers apoptosis in T lymphocytes, accompanied by the release of damage-associated molecular patterns.^19^ Apoptosis occurs preferentially in activated alloreactive T cells within 24 h, whereas resting T cells and monocytes require longer time frames—2 d and up to 6 d.^20,21^ Notably, although monocytes undergo delayed apoptosis, their functional properties remain largely intact.^21^ Monocytes play a central role by processing apoptotic antigens and interacting with circulating platelets. In parallel, platelet activation within the ECP circuit promotes monocyte-to-DC differentiation via P-selectin–dependent interactions.^22-25^ As a result, ECP facilitates the emergence of functional DCs from peripheral blood monocytes.^26,27^ DCs are central regulators of immune responses and possess tolerogenic potential.^28^ On phagocytosis of apoptotic cells, DCs can acquire a tolerogenic phenotype, characterized by the induction of regulatory T cells and the secretion of anti-inflammatory cytokines such as interleukin (IL)-10, IL-4, and transforming growth factor-beta (TGF-β).^29-31^ In parallel, 8-MOP has been shown to upregulate immunoregulatory genes, further supporting the generation of tolerogenic DCs.^32^ Furthermore, early studies reported increased tumor necrosis factor-alpha and IL-6 levels associated with the expansion of CD36^+^ macrophages.^33,34^ After reinfusion of treated cells, a shift toward an anti-inflammatory cytokine profile occurs, with elevated levels of IL-10, IL-4, and TGF-β and decreased levels of inflammatory IL-12, interferon alpha, tumor necrosis factor-alpha, and IL-1. This shift contributes to a Th1-to-Th2 polarization and expansion of regulatory T cells, which play a key role in suppressing immune activation and maintaining self-tolerance.^35,36^

## HISTORICAL OVERVIEW OF ECP APPLICATIONS IN LTx

Since the early 1990s, ECP has been used to treat bronchiolitis obliterans syndrome (BOS), the earliest defined form of chronic rejection, in LTx recipients who do not respond to the augmentation of immunosuppressive therapies.^37-43^ Although immunosuppressive regimens are generally effective in mitigating the risk of acute rejection, a small subset of patients with resistant acute cellular rejection (ACR) do not respond to conventional treatments, requiring alternative solutions. The effectiveness of ECP in the prevention of acute rejection episodes was first demonstrated in the context of heart transplantation in a pilot study by Barr et al,^44^ where 60 patients were randomly assigned to receive photopheresis in addition to standard triple-drug immunosuppressive therapy (cyclosporine, azathioprine, and prednisone). After 6 mo of follow-up, the addition of photopheresis to the standard immunosuppressive regimen significantly reduced the risk of cardiac acute rejection without increasing the incidence of infection.^44^ This study significantly contributed to the application of ECP in solid organ transplantation. In a few studies after LTx, ECP has been indicated as a form of rescue therapy in cases of recurrent or ongoing ACR, resulting in the resolution of acute rejection episodes.^40,45^ Additionally, ECP has been used as prophylactic therapy in combination with standard triple immunosuppression, leading to a significantly lower incidence of ACR episodes and a significant reduction in infectious complications after LTx.^16^ ECP has also been used in a limited number of patients with AMR after LTx. Our center introduced ECP as an add-on therapy for clinical AMR with the ultimate purpose of preventing the rebound of DSA and stabilizing lung function.^46^ Despite clinical experience across various transplant settings showing promising results, much of the data are derived from single-center retrospective studies, often lacking proper untreated control groups. Another key limitation is the significant variability in patient responses to ECP. Clearly, more data and well-designed studies are needed to guide treatment decisions and improve outcomes after LTx. Ideally, high-quality randomized controlled trials (RCTs) are necessary to better demonstrate the efficacy and safety of ECP in LTx.

## ECP FOR CLAD

CLAD is identified by a persistent reduction in forced expiratory volume in 1 s (FEV₁) of ≥20% from the baseline value, which persists for a period of 3 mo. CLAD is further classified into stages 0–4 based on FEV₁ changes.^3,47^ CLAD can manifest in 2 main phenotypes: BOS, characterized by peribronchiolar immune-related injury and fibrosis, and restrictive allograft syndrome (RAS), characterized by progressive pleural and parenchymal fibrosis.^3^ In a mixed phenotype, both phenotypes can be present at the same time and patients can switch from one phenotype to the other.^47^ Currently, treatments of CLAD are limited. Augmentation of immunosuppressive therapy and the addition of azithromycin are the most broadly used.^48^ Other therapies such as ATG, orthoclone (monoclonal anti-CD3 antibodies), alemtuzumab, methotrexate, cyclophosphamide, and total lymphoid irradiation are used in progressive CLAD.^49^ For CLAD, particularly in patients with BOS unresponsive to previous interventions, the macrolide antibiotic azithromycin remains the most extensively studied option, although its impact on improving lung function is generally poor.^48^ ECP treatment has been used as second-line therapy for CLAD,^48,50^ and its effectiveness in improving organ survival and stabilizing FEV_1_ without any significant side effects is mostly documented by retrospective or observational studies.^41-43,51-58^ After a few case series in the early 2000s, the first single-center study showing ECP as a promising therapy of CLAD was published by Morrell et al.^43^

In this study, 60 patients with progressive BOS were started on ECP and registered a stabilization or even an improvement of FEV_1_. Our group published a prospective interventional study including 51 patients with BOS, unresponsive to azithromycin therapy.^42^ In this study, we observed that 61% of patients responded to the treatment showing sustained stabilization of the lung function after 6 mo of ECP therapy. The ECP responders not only had notably higher survival rates but also a reduced need for retransplantation compared with nonresponders. Few studies tried to identify predictors for response. Greer et al^59^ found that patients with RAS, as well as patients whose lung function deteriorated rapidly, had lower response rates and worse long-term outcomes in 65 patients with progressive CLAD. Similarly, in another analysis, only BOS was associated with better outcomes.^57^ Recently, the Hannover group proposed a new approach to assessing outcomes of patients with CLAD using a temporal characterization of allograft function. The authors suggested that grafts with lower performance at the beginning of ECP were more likely to be associated with worse outcomes.^59^ Recently, a European multicenter retrospective analysis, including Hannover, Pavia, and our center, presented long-term outcomes of ECP as a treatment for CLAD in the largest cohort published to date, including 631 LTx patients.^58^ The study showed that 63% of patients with CLAD experienced stabilization or improvement of allograft function after ECP initiation, and this was associated with a survival benefit. RAS was identified as the only predictor of mortality, whereas FEV_1_ at ECP initiation was a protective factor, suggesting that early use of ECP could be beneficial in terms of graft and patient survival.^58^ One prospective RCT assessing ECP efficacy as a treatment for CLAD is ongoing in the United Kingdom. The E-CLAD UK trial (ISRCTN 10615985) will include 90 patients, who will all receive standard of care for CLAD for 24 wk. Additionally, half of these patients will be randomized to receive further ECP treatment. The trial has already recruited patients, and results are expected in the coming years.

## ECP FOR STEROID-REFRACTORY ACR

The standard first-line treatment for ACR after LTx is high-dose corticosteroid pulse therapy, which is generally effective and well tolerated in most patients.^60^ However, cases of steroid-refractory ACR have been reported, and multiple studies have also shown that this is a significant risk factor for the development of BOS.^60^ Therefore, there is an urgent need to identify therapeutic approaches that can effectively treat steroid-refractory ACR. In 1995, Andreu et al^45^ were among the first in France to use ECP for LTx, treating a patient with acute rejection and severe infection.^45^ After 3 wk of combined anti-infectious therapy and ECP, the patient showed clinical improvement, and histological signs of acute rejection disappeared after 4 wk. Villanueva et al^40^ later reviewed ECP use in 14 LTx patients with BOS. In 3 cases where BOS was accompanied by acute rejection, ECP resolved the acute rejection.^40^ Since the publication of early studies, ECP has been successfully used in ACR cases. In 2008, a single-center study demonstrated that ECP not only slowed the decline of lung function in patients with BOS but also stabilized those with recurrent ACR. In this study, the original cohort was followed up further 5 y, including 21 of the original 24 patients, with 9 treated for CLAD and 12 for recurrent ACR. Results indicated better survival in the ACR group compared with the CLAD group (82% versus 66%).^61^ Although ECP has shown promising results as a rescue therapy for recurrent or ongoing ACR, robust evidence is still lacking.

## ECP FOR AMR

Traditionally, AMR has been described as driven by dnDSAs that target HLA class I or II molecules on graft endothelium, triggering the classical complement pathway and leading to immune responses that damage the lung allograft, ultimately causing graft injury and dysfunction.^62,63^ Unfortunately, the current diagnostic criteria, including allograft dysfunction, histological signs of tissue injury, C4d staining, and circulating DSAs, are not sensitive enough, showing positivity in only 40%–60% of cases. Also, the treatment of AMR remains a subject of debate, with current management approaches primarily based on case reports and small single-center studies, and overall outcomes remaining very poor.^7,64-71^ During the past few years, increasing attention has been focused on subclinical AMR, particularly in patients with DSAs and no clinical signs of allograft dysfunction. Indeed, associations between dnDSAs and worse outcomes have been extensively described.^5,6,8,63,72-75^ To corroborate the subclinical impact of dnDSAs on the immunologic fate of the graft, depletion of dnDSAs or inactivation of their complement-binding activity was associated with better outcomes. A study by Witt et al^70^ found that patients who successfully cleared dnDSAs had better survival rates than those with persistent antibodies. In a single-center cohort study involving 340 LTx patients, individuals with preexisting DSAs who underwent antibody-directed desensitization before transplantation had similar 1-y outcomes compared with nonsensitized patients.^76^A prospective cohort study by Hachem et al^77^ showed comparable rates of CLAD and acute rejection in patients who developed DSAs and received antibody-directed therapy and those who did not develop DSAs. Additionally, a multicenter retrospective analysis by Keller et al^78^ reported that asymptomatic patients with dnDSAs who received preventive treatment had a lower risk of CLAD or death compared with those who did not receive treatment. Although these studies hint at the potential benefit of addressing dnDSAs in the absence of lung allograft dysfunction, the available treatments, including B cell–depleting antibodies, proteasome inhibitors, plasma cell–depleting antibodies, and plasma exchange, are associated with significant side effects, which limit their usability in the subclinical setting. Given its favorable safety profile and low incidence of adverse events, ECP may represent a viable option for treating asymptomatic dnDSA-positive patients. Specifically, ECP may promote tolerance to donor lung antigens by modulating antigen presentation and suppressing the generation of antibody-producing plasma cells, thereby representing a valuable, safe, and effective option in subclinical AMR.

ECP has only been used in a limited number of patients with AMR after solid organ transplantation. In a series involving 4 heart transplant recipients with elevated DSAs, prophylactic ECP leads to a reduction in circulating antibodies and a low incidence of rejection events.^79^ A multicenter retrospective study included 33 kidney transplant recipients who were treated with ECP for allograft rejection (23 AMRs, 2 chronic AMRs, and 8 ACRs).^80^ At 12 mo after ECP, 11 patients had stabilized kidney function with a graft survival rate of 61%. Patients with graft loss at 12 mo tended to have ACR and/or AMR, higher serum creatinine levels, DSA levels, and histologic scores of AMR.

It is estimated that 20%–50% of LTx recipients develop dnDSAs within the first year posttransplantation,^81^ and these patients generally exhibit reduced survival rates and a higher probability of developing CLAD.^4-8^ Furthermore, persistent dnDSAs, despite antibody-depleting treatments, have been linked to poorer outcomes.^82^ Therefore, strategies aimed at depleting dnDSAs or blocking their complement-binding activity have been associated with improved prognosis.^83^

After LTx, our center reported the first experience with ECP as an add-on treatment for clinical AMR, showing clearance or reduction of DSAs in most patients.^46^ In our study, in 14 patients, dnDSAs were cleared during ECP treatment; however, 6 of them developed CLAD, and only 26% of patients were still alive after 5 y. This suggests that the acute injury typical of AMR could be irreversible despite DSA clearance. In a study investigating the effectiveness of ECP in clearing DSAs in patients with BOS, 19 patients (70%) showed a decrease or stabilization of DSAs during the ECP treatment.^84^ All patients, however, received concomitantly IVIGs and 18 of them rituximab.^84^ Currently, a prospective RCT led by the Vienna Lung Transplant Program (EXPORT-DSA study, NCT06112951) is ongoing to evaluate the efficacy of ECP in subclinical AMR and to prevent the development of CLAD. In this study, 80 patients with persistent dnDSAs (>3 mo), with an Mean fluorescence intensity of >1000 and no signs of allograft dysfunction, will be randomized 1:1 into 2 groups. Patients are stratified on the basis of the presence of HLA-DQ DSAs. The control group is not receiving any active treatment. The treatment group receives ECP. First, a 2-d treatment cycle is performed once every second week for the first 2 mo. Then, a 2-d treatment cycle is performed once per month for 6 mo. The primary endpoint of the study is defined as the change in mean fluorescence intensity from baseline to the end of the therapy. To elucidate the effects of ECP on modulating humoral alloresponse, a plethora of experimental assessments, including immunophenotyping, cytokine expression, gene expression signatures of peripheral blood mononuclear cells and proteomic characterization as well as in vitro studies, have been performed. The results of this study are expected within 3 y and should provide valuable insights into the role of ECP in controlling humoral immunity.

## ECP AS PREVENTIVE THERAPY

Overall, ECP is currently used as a rescue therapy for refractory ACR, an adjunct treatment for AMR, and in cases of CLAD, where it has shown notable responses. A single prospective randomized study performed on heart transplant recipients by Barr et al^44^ in 1998 showed a significant reduction in cardiac rejection rates without increasing the incidence of infections by adding preventive ECP to a triple-drug immunosuppressive therapy. This older study constituted the rationale for the recently published prospective, randomized single-center trial performed at our institution. Our trial aimed to evaluate the safety and efficacy of adding ECP to standard immunosuppression for preventing ACR in the first year after LTx.^16^ In this trial, 62 adult recipients at the Vienna Lung Transplant Program (Medical University Vienna) were randomly assigned to either standard triple immunosuppressive therapy (tacrolimus, mycophenolate mofetil, and prednisone) or the same regimen with the addition of ECP. Results demonstrated that ECP significantly reduced the incidence of ACR and infection-related complications, leading to a reduced need for aggressive rejection treatments (eg, high-dose steroids or ATG). Moreover, a reduction of CLAD at 24 mo was observed in the ECP group.^16^

## FUTURE PERSPECTIVES

The exact mechanisms by which ECP affects the immune system remain unclear; however, previous research involving experimental models,^85^ in vitro assays, and preliminary human studies has proposed several mechanisms.^86^ In a BOS experimental model, ECP was found to reduce fibrogenesis driven by TGF-β, potentially explaining its observed benefits in preventing or stabilizing graft fibrosis in LTx.^42^ Another effect of the ECP may be the suppression of the T cell–dependent antibody production from memory B cells and plasmablasts,^87^ through the induction of regulatory T and B cells and by inhibiting IL-6 production.^88-90^ Supporting this, research by Li et al^91^ identified lung allograft-resident FOXP3^+^ cells in bronchus-associated lymphoid tissue as key negative regulators of allogeneic humoral responses. ECP has also been shown to increase IL-10^+^ regulatory B cells while depleting circulating allo- and autoreactive B-cell clones, leaving only 10% of CD19^+^ B cells viable posttreatment.^89,92^ Additionally, ECP induces tolerogenic myeloid-derived suppressor cells and reduces natural killer cell activity.^89,93^ An epigenetic study found that patients with BOS who responded to ECP after LTx showed an increase in miR-155-5p levels after 6 mo of treatment. Previous research suggests that the upregulation of miR-155 may be linked to protolerogenic immune system modulation, but further longitudinal studies are needed to explore the potential role of miR-155 and its downstream targets.^94^ Nevertheless, further research is required to validate these findings and assess whether ECP can be effectively integrated as a treatment for chronic graft fibrosis and acute rejection in LTx.^95^

## CONCLUSIONS

The studies reported that ECP can reduce DSAs, minimize acute rejection episodes, and either improve or stabilize FEV_1_ in patients with CLAD, with minimal side effects or toxicities, especially when initiated at early stages in the transplant journey. Patient responses to ECP vary significantly, and more research is necessary to identify reliable predictors of response.^96^ High-quality RCTs are needed to answer these open questions.

## OVERVIEW OF REVIEWED STUDIES

A summary of all studies discussed in this review, including the number of patients enrolled, clinical indications, and key findings of the use of ECP in transplantation, is presented in Table 1.

**TABLE 1.** Summary of studies evaluating the use of ECP in transplantation

Study (author, year)	Indication	No. of patients	Key findings
Benazzo et al, 2025^16^	Preventive (ACR and CLAD)	62 (RCT)	ECP reduced ACR, infections, and CLAD incidence at 24 mo
Villanueva et al, 2000^40^	BOS + ACR	14 (3 with ACR)	ECP resolved ACR in patients with BOS
Morrell et al, 2010^43^	CLAD (BOS)	60	ECP led to FEV_1_ stabilization/improvement in patients with BOS
Barr et al, 1998^44^	ACR (heart transplant)	60	ECP reduced cardiac ACR; foundational study supporting immunomodulatory role
Andreu et al, 1995^45^	Steroid-refractory ACR	1	Resolution of ACR and infection after ECP
Slovis et al, 1995^37^	CLAD (BOS)	3	Early report of ECP use in BOS
O'Hagan et al, 1999^38^	CLAD (BOS)	5	ECP for refractory BOS
Salerno et al, 1999^39^	CLAD (BOS)	8	ECP as adjuvant in BOS
Benden et al, 2008^41^	CLAD (BOS)	24 (BOS n = 12, recurrent AR n = 12)	10-y single-center experience showed favorable outcomes
Jaksch et al, 2012^42^	BOS	51	61% patients with BOS responded with stable FEV_1_
Benazzo et al, 2020^46^	AMR	14	DSA clearance in most patients; 26% 5-y survival; 6 developed CLAD
Vazirani et al, 2021^51^	CLAD	12	Positive outcomes after ECP in patients with BOS
Pecoraro et al, 2017^52^	CLAD	239	Patients with BOS benefitted from ECP
Moniodis et al, 2018^53^	CLAD	267	Compared ECP vs alemtuzumab in CLAD
Leroux et al, 2022^54^	CLAD	25	Real-life ECP experience in mild/moderate BOS
Karnes et al, 2019^55^	CLAD (BOS)	60	Identified mortality and response predictors in patients with BOS
EPI study Group, 2021^56^	CLAD	44	ECP attenuated FEV_1_ decline
Del Fante et al, 2015^57^	CLAD	48 patients with CLAD treated by ECP compared with 58 controls	10-y experience in BOS; long-term stabilization
Hannover-Pavia-Vienna multicenter, 2023^59^	CLAD (BOS and RAS)	631	63% had stabilization/improvement; survival benefit noted; RAS = poor prognosis predictor.
Greer et al, 2023^59^	CLAD	65	BOS responded better to ECP than RAS; lung function at ECP start predicted outcomes.
E-CLAD-UK Trial (ongoing)	CLAD	90 (planned)	RCT assessing ECP efficacy vs SoC; results awaited.
Witt et al, 2013^70^	AMR	340	Reduction in dnDSAs correlated with better survival
Rose et al, 1992^79^	AMR (heart transplant)	4	ECP lowered DSA, reduced rejection
Tamain et al, 2019^80^	AMR + ACR (kidney Tx)	33 (23 AMRs, 8 ACRs)	61% graft survival at 12 mo; DSA reduction with ECP
Baskaran et al, 2014^84^	AMR (BOS with DSA)	27	70% showed DSA stabilization or reduction during ECP + IVIG + rituximab
EXPORT-DSA Trial (ongoing)	AMR (subclinical dnDSA)	80 (planned)	RCT evaluating ECP effect on dnDSA clearance and prevention of CLAD

ACR, acute cellular rejection; AMR, antibody-mediated rejection; AR, acute rejection; BOS, bronchiolitis obliterans syndrome; CLAD, chronic lung allograft dysfunction; dnDSA, de novo donor-specific antibody; ECP, extracorporeal photopheresis; FEV_1_, forced expiratory volume in 1 s; RAS, restrictive allograft syndrome; RCT, randomized controlled trial; SoC, standard of care.
